# Epilepsy and Neuroscience: Evolution and Interaction

**DOI:** 10.3389/fnana.2020.00025

**Published:** 2020-06-12

**Authors:** Edward Henry Reynolds

**Affiliations:** Department of Clinical Neurosciences, King’s College, London, United Kingdom

**Keywords:** neuroscience, history, neurology, psychiatry, epilepsy

## Abstract

Neuroscience is a relatively new and fashionable word that emerged in the 1950s in several countries, including the UK, to describe a multidisciplinary clinical and laboratory approach to the study of the brain, mind, and neuropsychiatric disorders. However collaborative study of neurological and psychiatric disorders can be traced to the 17th century with roots in antiquity. I describe the evolution of our understanding of epilepsy beginning with the first detailed clinical descriptions, associated with supernatural theories, in Babylonian medicine in the second millennium BC. Interest in natural causation arose in the Greco-Roman period when it was first suggested that “the sacred disease” was a disorder of the brain. However, this theory did not take root until the 17th and 18th centuries AD when epilepsy began to be separated from other “convulsive” diseases, including hysteria. In the 19th century developments in neuropathology and our understanding of cortical localization led to the much-debated separation of idiopathic from symptomatic epilepsy which continues to influence international classifications of seizures and epilepsies. Also in the 19th century, the concept of seizures as electrical discharges in the brain evolved, reinforced in the 20th century by the discovery of the electroencephalogram. For many reasons, people with epilepsy have experienced a high incidence of cognitive and psychosocial disorders. Epilepsy, which is a global problem, has, therefore, remained a bridge between neurology and psychiatry. Furthermore, the study of epilepsy continues to shed light on brain function and other neuropsychiatric disorders.

## When Did Neuroscience Begin?

Neuroscience is a relatively new and fashionable word to describe a collaborative or multi-disciplinary approach, both clinical and laboratory, to the study of the nervous system, especially brain and mind, and their diseases. The word and concept evolved in several countries in the 1950s, as illustrated in Shepherd’s (2010) book “Creating Modern Neuroscience.”

In the UK, for example, collaborative neuroscience began in 1949 with Richter’s Research Unit at Whitchurch Hospital, Cardiff, which evolved in the 1950s into the MRC Neuropsychiatry Research Unit (Richter, 1972). Richter, with many others, contributed to the foundation of the International Brain Research Organisation (IBRO) in 1961 and of which he later became Secretary-General. He also played the leading role in founding the UK Brain Research Association in 1968, which in 1997 changed its name to the British Neuroscience Association (Reynolds, 2017).

Shepherd included the word “modern” in the title of his book because a collaborative approach to the brain, mind, and diseases of the nervous system can be traced long before the 20th century. Even at the beginning of the 20th century, the International Brain Commission was established in 1903 and supported the development of several Brain Research Institutes in Europe, including one in the USA, before the whole initiative succumbed to the first World War in 1914 (Richter, 2000). The journal Brain was established in the UK in 1878 and remains the oldest continuous neurological journal. Neurology emerged from neuropsychiatry as an independent discipline in the middle of the 19th century, whilst neuropsychiatry itself evolved mainly from the 17th century onwards, but with roots that can be traced to antiquity (Spillane, 1981; Trimble, 1981; Reynolds, 1990).

A brief account of the evolution of neuroscience from antiquity would be challenging, but in this article, I will outline the evolution of our understanding of epilepsy as an example of the evolution of neuroscience.

## The Second Millennium BCE: Early Clinical Descriptions

The earliest descriptions of what we now call neurological and psychiatric disorders can be found in Babylonian medical texts originating in the first half of the second millennium BC. The Babylonians were remarkably astute and objective observers and describers of medical illness and human behavior. I have had the privilege of collaborating with James Kinnier Wilson, an Assyriologist at Cambridge University and the son of the distinguished neurologist Samuel Alexander Kinnier Wilson of Wilson’s disease fame, on Babylonian texts located in museums in London, Paris, Berlin, and Istanbul (Reynolds and Kinnier Wilson, 2014). The Babylonians provided remarkably detailed accounts of what we today call epilepsy, stroke, psychoses, obsessive-compulsive disorder (OCD), psychopathic behavior, depression, and anxiety (Reynolds and Kinnier Wilson, 2014). For example, they include most of the common seizure types we know today e.g., tonic-clonic, absence, focal motor, etc, as well as auras, post-ictal phenomena, provocative factors (such as sleep or emotion) and even a comprehensive account of schizophrenia-like psychoses of epilepsy. They also recorded that seven or more seizures in 1 day i.e., status epilepticus, could lead to death. The Babylonians described the unilateral nature of stroke, involving limbs, face, speech, and consciousness, and distinguished the facial weakness of stroke from the isolated facial paralysis we call Bell’s palsy (Reynolds and Kinnier Wilson, 2014). They also recorded an accurate description of an agitated depression with biological features including insomnia, anorexia, weakness, impaired concentration and memory. The obsessive behavior described by the Babylonians included such modern categories as contamination, the orderliness of objects, aggression, sex, and religion. Accounts of psychopathic behavior include the liar, the thief, the troublemaker, the sexual offender, the immature delinquent, and social misfit, the violent, and the murderer (Reynolds and Kinnier Wilson, 2014).

The first line of the Babylonian text on epilepsy states:

“If epilepsy falls once upon a person or falls many times, it is the result of possession by a demon or a departed spirit” (Reynolds and Kinnier Wilson, 2014).

The Babylonians did not doubt whether a single seizure is epilepsy. Every attack, whether single or multiple, was a result of possession. Here is a remarkable account, for example, of a left-sided focal motor attack, in which progression to loss of consciousness makes it harder to drive out the demon:

“If at the time of his possession, while he is sitting down, his left eye moves to the side, a lip puckers, saliva flows from his mouth, and his hand, leg, and trunk on the left side jerk like a newly slaughtered sheep, it is miqtu. If at the time of the possession his mind is consciously aware, the demon can be driven out: if at the time of possession his mind is not so aware, the demon cannot be driven out” (Reynolds and Kinnier Wilson, 2014).

A Babylonian description of status epilepticus states:

“If an epilepsy demon falls many times upon him and on a given day he seven times pursues and possesses him, his life will be spared. If he should fall upon him eight times his life may not be spared” (Reynolds and Kinnier Wilson, 2014).

Although today we would not regard the number seven as crucial in the prognosis of status epilepticus, to the Babylonians and other ancient cultures seven was a sacred number with supernatural significance.

The Babylonians had only a superficial knowledge of anatomy and no knowledge of the brain, spinal cord, or psychological function. They had no systematic classifications of their own and would not have understood our modern diagnostic categories. Some neuropsychiatric disorders e.g., stroke or facial palsy had a physical basis requiring the attention of the physician or *asu*, using a plant and mineral-based pharmacology. Most disorders such as epilepsy, psychoses, and depression were regarded as supernatural due to evil demons and spirits or the anger of personal gods, and thus required the intervention of the priest or *asipu*. Other disorders such as OCD, phobias, and psychopathic behavior were viewed as a mystery, yet to be resolved, revealing a surprisingly open-minded approach (Reynolds and Kinnier Wilson, 2014).

From the perspective of a modern neurologist or psychiatrist, these ancient descriptions of neuropsychiatric phenomenology suggest that the Babylonians were observing many of the common neurological and psychiatric disorders that we recognize today, including epilepsy. There is nothing comparable in the ancient Egyptian medical writings and the Babylonians, therefore, were the first to describe the clinical foundations of modern neurology, psychiatry, and neuroscience.

## The First Millennium BCE and First Millennium AD: Early Natural Causation

In the Greco-Roman period, clinical descriptions of neurological and psychiatric disorders were much more fragmentary compared to Babylonian accounts. But the Greeks and Romans had now evolved a primitive knowledge of neuroanatomy and a much greater interest in natural causation.

In the Corpus Hippocraticum, a collection of about 60 separate works from the Greek classical period of about 420 BCE to the Hellenistic period and perhaps beyond, brief descriptions of “Epilepsia,” invariably loss of consciousness with or without convulsions, can be found but they are overridden with much more detailed accounts of both physical interpretations and treatments (Temkin, 1971; Reynolds, 2018). But at least for Hippocrates and his School of physicians “The Sacred Disease” was now for the first time viewed as a disease of the brain (Loeb Classical Series, 1923–1995; Hippocrates, 1985).

“The brain is the seat of this disease, as it is of other very violent diseases.”

The Hippocratic School envisaged seizures arising not by demonic possession but by cold phlegm passing into warm blood where it congeals. Epilepsy is considered a brain disease influenced by heredity and environment, especially temperature and wind direction. Therefore, if in the appropriate season the patient can be rendered humid and dry or hot and cold by regimen the disease could be cured so long as it had not become chronic and too powerful for the treatment.

Many Roman physicians, notably the influential Galen, endorsed these concepts and both Greek and Roman physicians began the process of distinguishing true “Epilepsia” from other forms of loss of consciousness, notably “hysteria,” a new Greek concept of multiple medical symptoms, including loss of consciousness, attributed to a wandering uterus (Reynolds, 2018).

For example, in the first century AD Celsus, a Roman physician, states:

“From the womb of a woman, also there arises a violent malady; and next to the stomach this organ is affected the most by the body, and has the most influence upon it. At times it makes the woman so insensible that it prostrates her as if by epilepsy. The case, however, differs from epilepsy in that the eyes are not turned, nor is there foaming of the mouth, no spasm of sinews; there is merely stupor” (Celsus, 1935).

However, Greek and Roman physicians had little understanding of physiology or brain function. They introduced the concept of “vital spirits” produced in the left ventricle of the heart and distributed to all parts of the body. In the brain, they were converted into “animal spirits” or “psychic pneuma,” a spiritual life-force that was stored in the ventricles of the brain from where it was distributed to the nerves which mediated both movement and sensation (Spillane, 1981). Also, disease and even temperament were mediated by a complex imbalance of four postulated bodily humors, i.e., black bile, an excess of which could result in a melancholic personality or even melancholia; yellow bile associated with a choleric temperament; phlegm (phlegmatic) and blood (Sanquine; Simon, 1978).

Greek and Roman physicians and philosophers did however also introduce a new subjective dimension to their descriptions of disease. Whereas the Babylonian approach was anonymous and objective the Greeks presented personal case histories, often naming the individual and their residence. This may be because Greek philosophers appear to have been the first to grapple with the concept of identity and the seat of thought, reasoning, judgment, and emotions. Greek philosophers also introduced the concept of the immaterial “psyche” or soul and questions arose as to the seat of the soul as well as of intellectual functions? The debate is best illustrated by Plato (427–347 BCE) and his pupil Aristotle (384–322 BCE). Plato placed the immortal and irrational soul in the head or brain but Aristotle thought the brain, which was cold and insensitive, was only a cooling gland for the blood and that therefore the heart was the seat of human sensation and knowledge (Crivellato and Ribatti, 2007; Reynolds, 2018). These competing encephalocentric and cardiocentric theories were debated by numerous philosophers and physicians without agreement throughout the Greek and Roman civilizations and for many centuries thereafter. As late as 1628 AD Harvey (Harvey, 1628) stated that the heart was the source of sensitive and motor life.

It is important to note that these early and much-debated Greek and Roman insights into physical causation and treatment, including multiple herbal and animal remedies, were beacons of enlightenment in an otherwise desert of supernatural healing. Most patients had little access to these more rational understandings and probably not very effective treatments. Supernatural theories of epilepsy continued to dominate in all civilizations up to the 17th century AD (Temkin, 1971; Reynolds, 2018).

## The Seventeenth—Nineteenth Centuries: Epilepsy as a Nervous/Brain Disorder

According to Lopez-Pinero (1983), the concept of nervous diseases emerged in Britain in the 17th century with *Willis and Sydenham*, who, for example, viewed hysteria and hypochondria as nervous diseases and not, as in the Galenic tradition, vapors emanating from the uterus or the liver, spleen, and stomach. Willis (1684) initiated the gradual relocation over the next two or three centuries of hysteria from the uterus to the brain by stating: “So-called uterine disease is primarily a convulsive disease caused by an alteration of the nerves and the brain.” The concept of nervous diseases was consolidated in the 18th century, notably by Whytt (1765) who considered: “All diseases may, in some sense, be called affections of the nervous system, because in almost every disease the nerves are more or less hurt; and in consequence of this various sensations, motions and changes are produced in the body.”

As the Greek concept of epilepsy as a brain disorder gradually took root in Europe there was much uncertainty and debate as to what to include within the concept of “convulsive diseases.” Gradually hysteria, tremors, rigors, tetanus, and other paroxysmal neurological disorders were separated (Todd, 1849; Jackson, 1890). At the same time there began a prolonged debate about the distinction between generalized seizures, for example, tonic-clonic or absence (“petit mal”) and focal or partial seizures; and between the concepts of primary generalized epilepsy, in which the brain is macroscopically normal, and secondary symptomatic epilepsy associated with many different brain pathologies. These debates have continued well into the 20th century leading to the current International League against Epilepsy classifications of Seizures and Epilepsies, which are still being refined (Reynolds, 2009).

The above debates were influenced by two other major 19th-century neuroscientific developments. First, the earlier concept of “nervous diseases” was gradually separated by advances in neuropathology into those disorders with brain pathology and those without any pathology. This, in turn, led to the development of neurology as an independent discipline from the mid-19th century onwards. Second, concepts of epilepsy were also influenced by increasing understanding of cortical localization of function stimulated both by studies of speech, for example, Broca (1824–1880), and of epilepsy itself (Ferrier, 1876). Thus the discovery of the motor cortex (Fritsch and Hitzig, 1870) led to the concept of “epileptiform” or “partial” seizures as models for the study of “generalized” seizures (Jackson, 1870, 1890). By meticulously studying the clinical features of unilateral epileptiform motor seizures, Jackson was able to conclude (as was later confirmed experimentally) that the motor cortex was concerned with movements rather than individual muscles. Paroxysmal episodes of an intellectual, emotional, or behavioral kind, including hysteria or “hystero-epilepsy” (Gowers, 1881), were more difficult to classify and localize. It was not until the discovery of the human electroencephalogram in the 20th century (Berger, 1929) that the concepts of temporal lobe or frontal epilepsy were gradually clarified, and psychological concepts of hysteria evolved.

## Electrical Basis of Epilepsy

As the concept of a brain disorder was gradually established between the 17th and 19th centuries, it was initially widely believed that epilepsy must have a vascular basis attributable to either acute anemia or acute congestion of the brain (Temkin, 1971). Todd was the first to develop an electrical theory of brain function and epilepsy in his Lumleian Lectures (Todd, 1849). Todd was an anatomist, physiologist, and pathologist as well as an outstanding physician with an interest in disorders of the nervous system. Through his contact with his contemporary in London, Michael Faraday, Todd was aware of the great discoveries in electromagnetism. Influenced by Faraday, he conceived of “nervous force” as a polar force, analogous to electricity but mediated by unknown molecular and nutritional mechanisms. He, therefore, preferred the term “nervous polarity.” Applying Faraday’s concept of “disruptive discharge,” he viewed seizures as the result of electrical discharges in the brain. According to Todd: “These periodic evolutions of the nervous force which give rise in the complete epileptic paroxysm may be compared to the electrical phenomenon described by Faraday under the name ‘disruptive discharge’.” (Todd, 1849; Reynolds, 2007). This is the origin of our use of the word “discharge” in epilepsy today (Reynolds, 2004, 2009).

As summarized in Table 1 it took more than 80 years for Todd’s electrical concepts of brain function and epilepsy to take root. His views were supported by the work of Caton (1877) with his discovery of electrical potentials in the brains of animals and of Berger (1929) who discovered the human electroencephalograph in the 1920s. In 1935, at the second International Neurological Congress in London, vascular theories of epilepsy were finally laid to rest and electrical theories widely embraced, due to the work of Berger, Lennox, Penfield, and others (Reynolds, 2005, 2009).

**Table 1:** Milestones in the history of brain electrical activity and discharges.

Davy	1800s	Sodium, potassium, chlorine, calcium, magnesium.
Faraday	1830s	Electromagnetism, animal electricity.
Todd	1840s	Brain electricity, nervous polarity, discharges.
Jackson, Ferrier	1870s	Cortical localization and excitability.
Fritsch, Hitzig		
Caton	1870s	Animal cortical resting and sensory potentials.
Cajal, Golgi	1890s	Neuron doctrine (1906 Nobel prize).
Berger	1920s	Human electroencephalogram, discharges.
Hodgkin, Huxley	1950s	Ionic basis of neurotransmission (1963 Nobel Prize).

Hodgkin and Huxley won the Nobel prize in 1963 for their discovery of the ionic basis of Todd’s “nervous polarity” or neurotransmission; the relevant ions of sodium and potassium, central to their hypothesis, were the very ones that Humphrey Davy had discovered in the first decade of the 1800s. Sir Humphrey Davy (1778–1829) was the leading British scientist and Director of the Royal Institution in London in the early part of the 19th century. It was he who took on as a laboratory assistant Michael Faraday, who then rose to succeed and surpass him in the fields of chemistry and physics (especially electromagnetism), and who so influenced Todd who applied his ideas to the brain (Reynolds, 2009).

## The Twentieth Century: Modern Neuroscience

By the turn of the 20th century the study of nervous diseases had evolved into three disciplines with rather indistinct or controversial boundaries, i.e., (1) neurology rooted in neuroanatomy, neuropathology and neurophysiology; (2) general psychiatry responsible for the care of poorly understood mental diseases but with diminishing interest or confidence in the neuropsychiatric approach, and; (3) psychodynamic psychiatry rooted in new psychoanalytic theories (Clarke and Jacyna, 1987; Reynolds, 1990). The place of epilepsy in neurology and psychiatry, however, remained uncertain and controversial. On the one hand, the growing evidence of electrical discharges in the brain underlying seizures and the association of epilepsy with many different brain diseases indicated that epilepsy was a neurological disorder. On the other hand patients with epilepsy, an intermittent and dramatic disorder, suffered profound psychological and social consequences, rooted in ignorance, misunderstanding, and stigma from antiquity. Many patients experienced anxiety, depression, and social isolation or rejection. Behavior disorders and even psychoses were not uncommon. Emotional factors had been known since Babylonian times to trigger seizures. Furthermore, most patients, especially children and adolescents, had no evidence of any brain pathology. In clinical practice, therefore, patients with epilepsy fell into the province of either neurology or psychiatry, or more appropriately, but rarely, into the diminishing field of neuropsychiatry, depending on the availability of limited services.

In the event, it took until the 1960s for the WHO to classify epilepsy as a neurological disorder. In the latter half of the 20th century, there has been a steady growth of neuroscientific interest in basic mechanisms underlying seizures and different age-dependent epilepsy syndromes, stimulated by developments in genetics, molecular biology, neurophysiology, neuropsychology, structural and functional imaging, and new techniques for exploring neurotransmission, excitation, inhibition, modulation, etc. At the same new diagnostic techniques such as electroencephalography, video telemetry, CT/MRI, neuropsychology, and pre-surgical evaluation have greatly improved the potential for accurate diagnosis and classification of seizures and epilepsies. There has also been a steady growth throughout the century in the availability of effective, but imperfect antiepileptic drugs, mostly of uncertain mechanisms and with varying undesirable side effects but each generating much neuroscientific interest (Reynolds, 2009).

Simultaneously there has been much interest in the mechanisms underlying cognitive, mental and behavioral disorders associated with epilepsy, including the varying roles of seizure discharges, brain pathology and neurophysiology, acute and chronic antiepileptic drug therapy, genetics, psychological and social determinants (Reynolds and Trimble (1981).

Epidemiological studies indicate that epilepsy is universally distributed around the world, affecting all ages, races, cultures, social classes, and countries (Engel and Pedley, 2008). We also know from studies of convulsive therapy for psychiatric disorders that given a sufficiently powerful stimulus all brains are capable of a convulsive response although with varying degrees of threshold. Every advance in our clinical and basic understanding of the epilepsies and their associated cognitive and psycho-social disorders seems to add to the enormous complexity of the nervous system and the probability that multiple elusive genetic-molecular-metabolic mechanisms contribute to the wide range of epilepsies and their complications. Indeed there is some evidence that epilepsy, which can occur in all mammalian species, does so more frequently as brains have become more complex (Servit, 1962). Perhaps the very complexity of the human brain increases vulnerability.

## The Influence of Epilepsy on Neuroscience

In the late 20th century there has been a convergence of the interests of neurologists and psychiatrists and a renewal of neuropsychiatry as an independent discipline, at least in the UK and some other Western countries where the divergence has been greater than in many other countries. The study of epilepsy has played a leading role in this process.

For example, Todd’s (1849) clinical and experimental studies of epilepsy, influenced by Michael Faraday, led to his seminal concept of nervous polarity and “electrical discharges” underlying seizures. This opened the door to the whole field of the neuronal and electrical basis of nervous system function (Reynolds, 2004; Binder et al., 2011).

Jackson’s (1870) detailed clinical studies of unilateral seizures, especially focal motor (Jacksonian) attacks, influenced his colleague David Ferrier to undertake his famous experimental studies of cortical electrical stimulation and of ablation, which led, among others, to our modern understanding of cortical localization (Ferrier, 1876) and, as already mentioned, to the concept of the localization of movements in the motor cortex.

The introduction of convulsive therapy for mental illness flowed in 1937 from Von Meduna’s (1937) clinical studies in Hungary of the effects of seizures on people with epilepsy and people with psychiatric disorders. This, in turn, led in Zurich to Landolt’s (1958) concept of “forced normalization” of the EEG and biological antagonism between seizures and some forms of mental illness.

Penfield and Jasper’s (1954) studies of cortical stimulation in epileptic patients undergoing pre-surgical evaluation led to new concepts of the dynamic function of the cerebral cortex. It was Penfield who reinforced the concept of epilepsy as “a great teacher” and a window on the brain.

Epilepsy has continued to be a bridge between neurology and psychiatry (Reynolds and Trimble, 1989) and there is little doubt that the evolving neuroscientific study of epilepsy will continue to shed light also on brain function and other brain and mental disorders.

## Author Contributions

The author confirms being the sole contributor of this work and has approved it for publication.

## Conflict of Interest

The author declares that the research was conducted in the absence of any commercial or financial relationships that could be construed as a potential conflict of interest.

## References

[B1] Berger H. (1929). Über des elektrenkephalogramm menschen. Arch. Psychiatr. Nervenkr. 87, 527–570.

[B2] Binder D. K. Rajneensh K. F. Lee D. J. Reynolds E. H. (2011). Robert Bentley Todd’s contribution to cell theory and the neuron doctrine. J. Hist. Neurosci. 20, 123–134. 10.1080/0964704X.2010.496611 21480036

[B3] Caton R. (1877). Interim report on investigation of the electric currents of the brain. BMJ 1, 62–65.

[B4] Celsus A. C. (1935). De Medicina. Eds. J. E. Page, E. Capps, and W. H. D. Rouse. Translated by W. G. Spencer. London: William Heinemann.

[B5] Clarke E. Jacyna L. S. (1987). 19th Century Origins of Neuroscientific Concepts. Berkeley, CA: University of California Press.

[B6] Crivellato E. Ribatti D. (2007). Soul, mind, brain: greek philosophy and the birth of neuroscience. Brain Res. Bull. 71, 327–336. 10.1016/j.brainresbull.2006.09.020 17208648

[B7] Engel J. Pedley T. A. (Eds). (2008). Epilepsy: The Comprehensive Textbook. Philadelphia, PA: Lippincott, Williams and Wilkins.

[B8] Ferrier D. (1876). The Functions of the Brain. London: Smith, Elder and Co.

[B9] Fritsch G. T. Hitzig E. (1870). Über die elektrische erregbarkeit des grosshirns. Arch. Anat. Physiol. Wiss. Med. 37, 300–332.

[B10] Gowers W. R. (1881). Epilepsy and other Chronic Convulsive Diseases. London: J & A Churchill.

[B11] Harvey W. (1628). Exercitatio Anatomica de Motu Cordis et Sanguinis in Animalibus. Frankfurt Am Main: Guilielmi Fitzeri.

[B12] Hippocrates (1985). “The sacred disease,” in The Genuine Works of Hippocrates: The Classics of Medicine Library (Birmingham, AL: Gryphon Editions), 843–858.

[B13] Jackson J. H. (1870). A study of convulsions. Trans St. Andrews Med. Grad. Assoc. 3, 162–204.

[B14] Jackson J. H. (1890). On convulsive seizures. Lancet 1, 685–688, 735–738, 785–788.

[B15] Landolt H. (1958). “Serial electroencephalographic investigations during psychotic episodes in epileptic patients and during schizophrenic attacks,” in Lectures on Epilepsy, ed. Lorentz de Haas A. M. (Amsterdam: Elsevier), 91–133.

[B16] Loeb Classical Series (1923–1995). Hippocratic Corpus, Volume 1—VII. London: William Heinemann.

[B17] Lopez-Pinero J. M. (1983). Historical Origins of the Concept of Neurosis. Translated by D. Berrios. Cambridge, MA: Cambridge University Press.

[B18] Penfield W. Jasper H. (1954). Epilepsy and the Functional Anatomy of the Human Brain. Boston, MA: Little, Brown & Co.

[B19] Reynolds E. H. (1990). Structure and function in neurology and psychiatry. Br. J. Psychiatry 157, 481–490. 10.1192/bjp.157.4.481 1983387

[B20] Reynolds E. H. (2004). Todd, Faraday, and the electrical basis of brain activity. Lancet Neurol. 3, 557–563. 10.1016/s1474-4422(04)00842-7 15324724

[B21] Reynolds E. H. (2005). The John Hughlings Jackson 1935 centenary congress medal. J. Neurol. Neurosurg. Psychiatry 76, 858–859. 10.1136/jnnp.2004.056184 15897511 PMC1739690

[B22] Reynolds E. H. (2007). Jackson, Todd, and the concept of “discharge” in epilepsy. Epilepsia 48, 2016–2022. 10.1111/j.1528-1167.2007.01162.x 17561951

[B23] Reynolds E. H. (2009). Milestones in epilepsy. Epilepsia 50, 338–342. 10.1111/j.1528-1167.2009.02050.x 19317878

[B24] Reynolds E. H. (2017). The origins of the British Neuroscience Association. Neuroscience 367, 10–14. 10.1016/j.neuroscience.2017.09.057 29066383

[B25] Reynolds E. H. (2018). Hysteria in ancient civilisations: a neurological review: possible significance for the modern disorder. J. Neurol. Sci. 388, 208–213. 10.1016/j.jns.2018.02.024 29525297

[B26] Reynolds E. H. Kinnier Wilson J. V. (2014). Neurology and psychiatry in babylon. Brain 137, 2611–2619. 10.1093/brain/awu192 25037816

[B27] Reynolds E. H. Trimble M. R. (1981). Epilepsy and Psychiatry. Edinburgh: Churchill Livingstone.

[B28] Reynolds E. H. Trimble M. R. (1989). The Bridge Between Neurology and Psychiatry. Edinburgh: Churchill Livingstone.

[B29] Richter D. (1972). Medical research council neuropsychiatry unit, carshalton and epsom, surrey. Psychol. Med. 2, 428–437. 10.1017/s0033291700045268 4571144

[B30] Richter J. (2000). The brain commission of the international association of academies: the first international society of neurosciences. Brain Res. Bull. 52, 445–457. 10.1016/s0361-9230(00)00294-x 10974483

[B31] Servit Z. (1962). Phylogenesis and ontogenesis of the epileptic seizure. World Neurol. 3, 259–274. 13910932

[B32] Shepherd G. M. (2010). Creating Modern Neuroscience: The Revolutionary 1950’s. Oxford: Oxford University Press.

[B33] Simon B. (1978). Mind and Madness in Ancient Greece: The Classical Roots of Modern Psychiatry. Ithaca, NY: Cornell University Press.

[B34] Spillane J. D. (1981). The Doctrine of the Nerves. Oxford: Oxford University Press.

[B35] Temkin O. (1971). The Falling Sickness, Second Edn. Baltimore, MD: The John Hopkins University Press.

[B36] Todd R. B. (1849). On the pathology and treatment of convulsive diseases. London Med. Gaz. 8, 661–671, 724–729, 766–772, 815–822, 837–846.

[B37] Trimble M. R. (1981). Neuropsychiatry. Chichester: John Wiley & Sons.

[B38] Von Meduna L. (1937). Die Konvulsions Therapie de Schizophrenie. Halle: Carl Marhold.

[B39] Whytt R. (1765). Observations on the Nature, Causes and Cure of those Disorders which are Commonly called Nervous, Hypochrondriac or Hysteric. Edinburgh: Beckett and Du Hondt.

[B40] Willis T. (1684). “Pathology of the brain and nervous stock: on convulsive diseases,” in The Remaining Medical Works of that Famous and Renowned Physician Dr Thomas Willis of Christ Church in Oxford, and Sidley Professor of Natural Philosophy in that Famous University, ed. Pordage S. (London: Dring, Harper Leigh and Martyn).

